# Integrative genetic and epigenetic control of skeletal muscle fiber traits in agricultural animals

**DOI:** 10.3389/fgene.2025.1566553

**Published:** 2025-05-02

**Authors:** Xiaolong Chang, Junwu Ma

**Affiliations:** National Key Laboratory of Pig Genetic Improvement and Germplasm Innovation, Jiangxi Agricultural University, Nanchang, Jiangxi, China

**Keywords:** agricultural animal, myofiber development, meat quality, regulation mechanism, epigenetic

## Abstract

Skeletal muscle fiber traits are fundamental to meat production and the meat quality of agricultural animals. The rich genetic resources and diverse phenotypic expression of muscle traits in agricultural animal species provide invaluable materials for investigating the genetic and molecular regulatory mechanisms underlying myofiber development and characteristics, optimizing breeding strategies, and developing models for human muscle-related diseases. This review presents an integrative perspective on the genetic and epigenetic regulation of skeletal muscle fiber development, incorporating evolutionary, genomic, epigenomic, and multi-omics insights. We focus on genetic architecture and causative or candidate genes for muscle fiber traits, as revealed by genome-wide association studies (GWAS) and selective sweep signatures, underscoring their adaptive significance and potential for selective breeding. The role of epigenetic mechanisms, such as DNA methylation, histone modifications, and non-coding RNAs, in linking genetic variation and phenotypic expression is also discussed. By synthesizing multi-omic data, we provide a comprehensive understanding of the molecular networks driving muscle fiber growth and differentiation. This review aims to consolidate current knowledge and offer actionable insights to advance research, breeding strategies, and applications in agricultural and biomedical fields.

## 1 Introduction

Despite ongoing efforts, hunger and food insecurity persist, with billions of individuals lacking access to nutritious, safe, and adequate food (UNICEF, 2024). With the global population projected to reach 9.7 billion by 2050 (UN 2015), the demand for food is anticipated to surge. Agricultural animals are pivotal in global food production (Davis and White, 2020), with meat serving as a crucial dietary protein source. By 2033, poultry meat is expected to constitute 43% of protein intake from all meat sources, trailed by pork, beef, and mutton. Moreover, meat is a vital provider of essential nutrients, including vitamins and minerals (Gehring, 2023). According to the Organization for Economic Cooperation and Development (Oecd, 2024)/Food and Agriculture Organization of the United Nations (FAO) (Oecd, 2024), it is projected that by the year 2033, there will be a global increase in the consumption of poultry, pork, beef, and sheep meat by 16%, 8%, 11%, and 16%, respectively. Furthermore, *per capita* meat consumption is anticipated to rise by 2%. This escalating demand, combined with heightened consumer expectations regarding meat quality, underscores the imperative to enhance the production efficiency of agricultural animals.

Skeletal muscle, which constitutes approximately 40% of an animal’s body weight (Erimbetov et al., 2019), is pivotal to meat production and performs essential functions in metabolism, movement, and energy storage. The development of skeletal muscle is governed by intricate interactions between genetic factors, such as regulatory genes and non-coding RNA, and environmental influences. These interactions determine critical muscle fiber traits, including number, diameter, and type, directly affecting meat yield and quality. Through natural selection and selective breeding, agricultural animals have acquired distinct characteristics that set them apart from humans and conventional model organisms. Their genetic diversity and economic importance make them valuable subjects for muscle development research. Notably, swine have emerged as prominent models for human medical research due to their genetic and physiological resemblances with humans, thereby propelling advancements in the domains of genetics, developmental biology, disease mechanisms, and xenotransplantation (Prather, 2013; Fang et al., 2024; Meyerholz et al., 2024; Peterson et al., 2024). Therefore, a comprehensive understanding of the genetic and regulatory mechanisms underlying skeletal muscle growth is imperative for enhancing meat production, optimizing breeding strategies, and contributing to advancements in medical science.

Recent advances in molecular biology techniques and high-throughput sequencing technologies have significantly expanded our knowledge of the genetic and epigenetic frameworks underlying muscle fiber traits in agricultural animals. This review provides a comprehensive overview of how genetic and epigenetic factors contribute to skeletal muscle growth and myofiber characteristics in agricultural animals from evolutionary, genomic, epigenomic, and multi-omics perspectives.

## 2 Muscle morphological diversity in agricultural animals and its impact on meat productivity and quality

Agricultural animals exhibit substantial genetic diversity, shaped by natural evolution and intensive artificial selection (Andersson, 2001; Koshkina et al., 2023). This diversity is vital for expressing economically significant and adaptive traits, as well as understanding molecular mechanisms behind muscle growth and development (Zhong et al., 2020). Unlike genetically uniform model organisms like mice, livestock and poultry display significant variation within and across breeds (Jiang et al., 2019; Jiang et al., 2018). For example, Chinese local pig breeds exhibit superior traits such as high fertility and excellent meat quality (Huang et al., 2020), while poultry and cattle breeds reflect diverse genetic and phenotypic outcomes from domestication and targeted breeding (Makarova et al., 2019; Wang et al., 2020; Bennett et al., 2008; García-Ruiz et al., 2016). In cattle, small effective population sizes due to rigorous selection enhance the detection of recessive deleterious mutations, which are less prevalent than in natural populations due to purification mechanisms (Kemper et al., 2012; Eyre-Walker, 2010). This genetic diversity not only underpins agricultural advancements but also offers valuable insights for human biomedical research.

Given the challenges associated with direct human disease studies, including the limitations in available samples, environmental constraints, and ethical considerations, the use of animal models is imperative for elucidating disease mechanisms. Agricultural animals like pigs, cows, and sheep surpass traditional models like mice due to their closer physiological and genetic alignment with humans (Muroya, 2022; Petersen, 2023). Pigs, in particular, are considered to be significant candidates for xenotransplantation, vaccine development, and modeling human development, owing to their anatomical and genomic similarities (Abdoli et al., 2018; Lunney et al., 2021). For instance, porcine models of Duchenne muscular dystrophy (DMD) replicate human clinical features more effectively than mice, with studies demonstrating gene restoration and phenotypic parallels (Perleberg et al., 2018; Yu et al., 2016). These advantages highlight the critical role of agricultural animals in biomedical research.

Beyond their biomedical value, the genetic and phenotypic traits of agricultural animals are pivotal for enhancing meat production and quality. Myofiber characteristics, including their number, size, type, and metabolism, play a fundamental role in determining meat yield and quality (Lefaucheur, 2010; Joo et al., 2013). A comprehensive understanding of these traits is imperative for the optimization of breeding and management practices, thereby ensuring the fulfillment of consumer demand for meat products of superior quality (Godfray et al., 2018; Kopler et al., 2023).

Myofiber traits vary due to factors like breed, sex, physiological stage, nutrition, and environment. For instance, Chinese local pig breeds show higher levels of type I, IIa, and IIx myofibers compared to foreign breeds (Kahane et al., 2001), while Turkish sheep demonstrate a link between fiber diameter and tenderness (Şirin et al., 2017). Dairy cows possess more oxidized fibers than beef cattle, reflecting metabolic differences (Schreurs et al., 2008), and ducks have higher myofiber counts but smaller fiber sizes than chickens (Kim et al., 2008). Generally, pigs and poultry favor fast-twitch fibers for rapid growth, whereas cattle and sheep rely on slow-twitch fibers for endurance, showcasing species-specific adaptations.

These muscle fiber traits directly impact meat quality and production efficiency, influencing attributes like color, pH, and tenderness (Ismail and Joo, 2017). Type I fibers’ myoglobin content affects color and flavor (Buckingham and Rigby, 2014; Matarneh et al., 2021; Mashima et al., 2019), while glycolytic fibers correlate with shear force (Kim et al., 2016; Park et al., 2024). Genetic analyses reveal strong links between fiber density and quality indicators such as eye muscle area and color (Larzul et al., 1997; Choi and Kim, 2009). Myofiber number drives postnatal growth and feed efficiency (Wigmore and Stickland, 1983), yet selection for traits like increased muscle mass can reduce fiber counts, risking quality declines (Felício et al., 2013). Balancing fiber number and size is thus essential for optimal meat production (Callaghan et al., 2020; Li et al., 2015). In general, a higher number of muscle fibers combined with an appropriately sized muscle fiber cross-sectional area can improve the final lean meat productivity and ensure normal meat quality, while increasing the number and area ratio of type I and IIA muscle fibers and reducing the number and area ratio of type IIB muscle fibers to achieve a balance between meat quality and yield. A deeper grasp of the genetic and biological underpinnings of these traits can steer breeding programs toward improved meat quality and efficiency.

## 3 Genetic signals of selection in muscle mass and fiber traits

Agricultural animals, subjected to prolonged natural selection and artificial breeding, have exhibited marked adaptive alterations in morphology, physiology, and behavior. These phenotypic modifications represent the genome’s response to environmental challenges and human intervention, offering a distinct research opportunity to explore how genetic variation influences phenotypic diversity. With recent advancements in genomics technology, scientists have been able to pinpoint genetic selection signals linked to key economic traits, facilitating a deeper understanding of the molecular underpinnings of muscle growth and development.

Leif Andersson et al. (Rubin et al., 2010) employed massively parallel sequencing to discern advantageous alleles and candidate mutations that have significantly influenced chicken domestication. Notably, a selective sweep in broiler chickens aligns with genes including *IGF1* and *TBC1D1*, which are implicated in growth, appetite, and metabolic regulation. In addition, Tan et al. (2024) found that the *SOX6* gene has a key regulatory role in broiler breast muscle traits and adaptation to artificial selection. These insights elucidate the genetic underpinnings of rapid muscle growth in contemporary broiler breeds. Zhou et al. (2018) pinpointed two mutations in Pekin duck genes linked to white feathers, augmented body size, and enhanced feed-conversion efficiency through a comparative genomic analysis across diverse duck breeds. One mutation in the *MITF* gene correlates with white feather coloration, while another distinct mutation might induce sustained expression of the *IGF2BP1* gene postnatally in Pekin ducks, thereby augmenting meat yield. Skeletal muscle consists of multiple heterogeneous cell populations whose interactions are critical for the maintenance of muscle homeostasis. Xu et al. (2023) analyzed neonatal skeletal muscle cell populations from wild boar, Duroc, and Laiwu pigs using single-cell RNA sequencing (scRNA-seq) to investigate the effects of artificial selection on muscle cell profiles. It was found that compared to wild boar, domestic pigs had significantly more fiber adipose-forming progenitor (FAP) cells and fewer myoblasts, while the proliferation rate of myogenic progenitor cells was higher, suggesting a greater muscle growth potential in domestic pigs. In addition, cross-species comparisons identified 186 shared active transcription factors, as well as some (e.g., *CREB3L1*, *TGIF1*, *NFIC*, and *CEBPZ*) gene regulatory networks that have been shown for the first time to have a potential role in muscle development. These results provide new perspectives for understanding the effects of artificial selection on the spectrum of muscle-resident cells. Furthermore, Liu et al. (2024) unveiled epigenetic mechanisms that account for disparities in muscle growth between eastern and western pig breeds. Their analyses suggest that artificial selection impacts DNA methylation and gene regulation, subsequently affecting muscle growth and meat quality. The research highlighted pivotal genes such as *GHSR* and *BDH1* in modulating skeletal muscle development and meat production. Specifically, the *GHSR* gene fosters myoblast proliferation but impedes their differentiation and fusion. A C>T mutation in its intronic region exerts an allelic genetic effect of 4.22 kg on carcass weight at 240 days of age. Concurrently, an insertion acts as a potential enhancer regulating the *BDH1* gene in eastern pigs, causing variances in myofiber proliferation and differentiation across breeds. BDH1 is a crucial rate-limiting enzyme in ketone metabolism and ATP synthesis. The insertion-triggered elevated expression of BDH1 may be instrumental in myofiber proliferation and differentiation.

Research into genomic selection signals in agricultural animals has unveiled numerous critical genes and mutations associated with muscle growth and development. These insights not only elucidate the role of genetic variations in shaping phenotypic diversity but also offer a scientific foundation for enhancing agricultural animal breeds and boosting the efficiency of meat production.

## 4 Genetic architecture of myofiber traits

Heritability, a significant concept in animal breeding, quantifies the proportion of phenotypic variation in a trait attributable to genetic factors (Visscher et al., 2008). Concerning myofiber traits in agricultural animals, heritability estimation serves as a critical metric for evaluating the potential of these traits for genetic selection. A comprehensive understanding of myofiber trait heritability can inform the development of more scientifically rigorous and effective breeding strategies, thereby enhancing meat yield and quality. In recent years, advancements in quantitative genetics and genomic technologies have led to increasingly precise estimates of myofiber trait heritability, thereby providing crucial reference points for genetic improvement programs.

Heritability estimates for muscle fiber traits demonstrate considerable variation across different agricultural animal species. For instance, a study (Li H. et al., 2024) on Gushi chickens reported low heritability for both leg muscle fiber density and diameter (ranging from 0 to 0.2), with moderate heritability observed for intramuscular fat (0.35). On the other hand, heritability estimates for fiber traits in Kashmiri Merino sheep varied between 0.189 and 0.300 (Ahmad et al., 2022), suggesting some potential for genetic selection within these traits. In the case of Large White pigs (Larzul et al., 1997), myofiber traits exhibited moderate to high heritabilities, such as a heritability of 0.46 for type I fiber percentage and up to 0.58 for type IIB fiber percentage.

Further studies showed that myofiber traits also have large genetic variation across breeds and populations. Yan et al. (2024) conducted an analysis of the muscle fiber types and meat quality traits in 318 pigs from four distinct Shanxia Changhei pig populations. They discovered that the heritability range for meat quality traits spanned from 0.06 (pH at 24 h) to 0.47 (shearing force), while the heritability for muscle fiber types varied between 0.04 and 0.4. Notably, the highest heritability was observed for total fiber density (0.40), whereas the lowest was for the percentage of type IIA fibers (0.04). These findings align with those of previous studies (Lee et al., 2022), which reported heritability values ranging from 0.13 to 0.55. Furthermore, Huang et al. (Huang et al., 2022) determined that the heritability of muscle fiber number is approximately 0.3–0.54, considered moderate, and noted a coefficient of variation as high as 18.64% for the total number of muscle fibers. In a study involving the White Duroc × Erhualian F2 resource population, Li W. et al. (2009) observed a substantial 3.6-fold difference between the minimum and maximum values of the total number of muscle fibers. These estimates imply that pigs and broilers, with moderate to high heritability, are prime candidates for selection to improve myofiber traits, potentially enhancing meat quality and yield efficiently. Conversely, the lower heritability in sheep suggests that selection may yield slower progress, requiring alternative strategies like crossbreeding to capitalize on hybrid vigor. The variability across studies underscores how species, population structure, and genetic background shape trait inheritance, necessitating customized breeding approaches.

**FIGURE 1:**
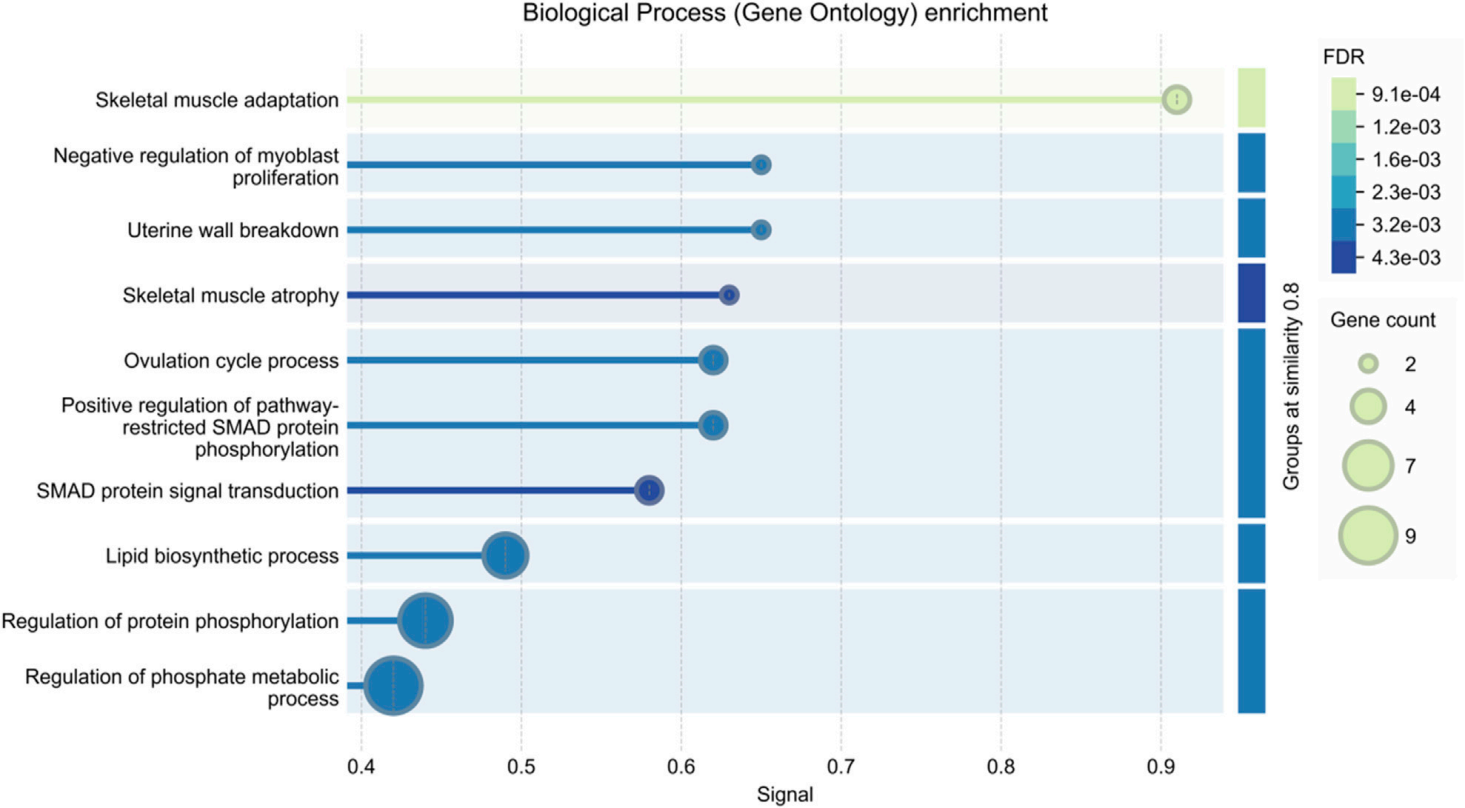
Enrichment analysis of biological pathways in quantitative trait loci (QTL)-associated genes for muscle fiber traits in agricultural animals.

The majority of myofiber traits in agricultural animals are controlled by multiple quantitative trait loci (QTL). There have been, and continue to be, successful attempts to localize QTL affecting myofiber traits (Wimmers et al., 2006; Guo et al., 2019). Table 1 provides a summary of the QTLs that impact the muscle fiber traits of pigs, cattle, sheep, and chickens as listed in the Animal QTLdb database. To date, there have been 12 documented QTLs that affect the total number of muscle fibers in pigs (https://www.animalgenome.org/cgi-bin/QTLdb/index, last updated on August 25, 2024). Further, we analyzed the biological pathway enrichment of these QTL-associated genes in Table 1 and found that they were significantly enriched in pathways related to muscle growth and development, as shown in Figure 1. In addition, Huang et al. (2022) conducted a genome-wide association study (GWAS) for nine myofiber traits using whole-genome sequence data from a heterogeneous population of eight breed crosses. Sixty-seven quantitative trait loci (QTL) for these traits were revealed, several loci were found to be significantly associated with the myofiber number phenotype, and some key candidate genes were identified. These results have facilitated the process of resolving the genetics of myofiber traits. However, despite the success of GWAS in identifying multiple loci, the heritability explained by these loci is usually lower than the total heritability estimate, a phenomenon known as “missing heritability” (Eichler et al., 2010; Trerotola et al., 2015). This discrepancy may stem from undetected factors such as epistatic interactions, rare variants, or epigenetic modifications. Current genomic tools, while powerful, struggle to capture these complexities, limiting the resolution of trait genetics. Addressing this requires advanced approaches like whole-genome sequencing, multi-omics integration, and the utilization of extensive, diverse datasets to elucidate the comprehensive genetic underpinnings of myofiber traits.

**TABLE 1:** Current status of QTL research on muscle fiber traits in pig, cattle, sheep, and chicken.

Species	Myofiber Traits	QTL Numbers	Associated genes	References
Pig	Total muscle fiber number	12	*BHMT*, MYOD1, *KNDC1*, MIR133B	Wimmers et al. (2006), Guo et al. (2019), Lee et al. (2012), Li et al. (2009b), Lee et al. (2013)
	Number of muscle fibers per unit area	23	BHMT, *MYOD1*, *hpo-5*, KATNB1, WDFY4, *KNDC1*	Wimmers et al. (2006), Guo et al. (2019), Lee et al. (2012), Li et al. (2009b), Zhang et al. (2020)
	Diameter of muscle fibers	11	*hpo-5*, *WDR47*, SLC44A5	Wimmers et al. (2006), Zhang et al. (2020)
	Diameter of type I muscle fibers	9		Wimmers et al. (2006), Estellé et al. (2008)
	Diameter of type IIa muscle fibers	7		Wimmers et al. (2006), Estellé et al. (2008)
	Diameter of type IIb muscle fibers	8	*hpo-5*	Wimmers et al. (2006), Estellé et al. (2008), Reiner et al. (2002)
	Cross-sectional area of muscle fibers	3	*MYOD1*	Lee et al. (2012)
	Cross-sectional area of type I muscle fibers	1		Li et al. (2009b)
	Cross-sectional area of type IIa muscle fibers	7		Li et al. (2009b)
	Cross-sectional area of type IIb muscle fibers	4		Li et al. (2009b)
Cattle	Muscle fiber diameter	3	HGD	Zhou et al. (2010)
	Muscle fiber area	1	*MSTN*	Allais et al. (2010)
Chicken	Muscle fiber cross-sectional area	2	FTO	Jia et al. (2012)
	Muscle fiber density	8	*HMGCR*, *LPIN2*, *LPIN1*, IL15, *TGFB3*, *PRKAG3*	Lv et al. (2012), Wei et al. (2012), Chen et al. (2013), Li et al. (2013), Huang et al. (2015)
	Muscle fiber diameter	10	*LPIN2*, *LPIN1*, *TGFB2*, IL15, *TGFB3*, PRDM16	Lv et al. (2012), Chen et al. (2013), Li et al. (2013), Huang et al. (2015), Han et al. (2012), Zhang et al. (2013)
	Muscle fiber number	2	*FIGF*, *AKT3*	Chen et al. (2013)
Sheep	Muscle density	12		Matika et al. (2016)
	Total muscle area	4		Matika et al. (2016)

*Note*. https://www.animalgenome.org/cgi-bin/QTLdb/index,last updated on August 25,2024.

Overall, muscle fiber traits in agricultural animals show different levels of estimated heritability, which highlights their potential application value in future genetic improvement programs, especially in improving meat quality and yield. However, the effective application of these traits in breeding practices requires a deep understanding of the species-specific genetic structure, as heritability and its trait expression can vary significantly among different livestock species. In addition, it is crucial to use advanced genomic technologies to develop breeding strategies tailored to these genetic differences to optimize meat production sustainably and efficiently.

## 5 The molecular regulatory network underlying skeletal muscle fiber development and phenotypes

**FIGURE 2:**
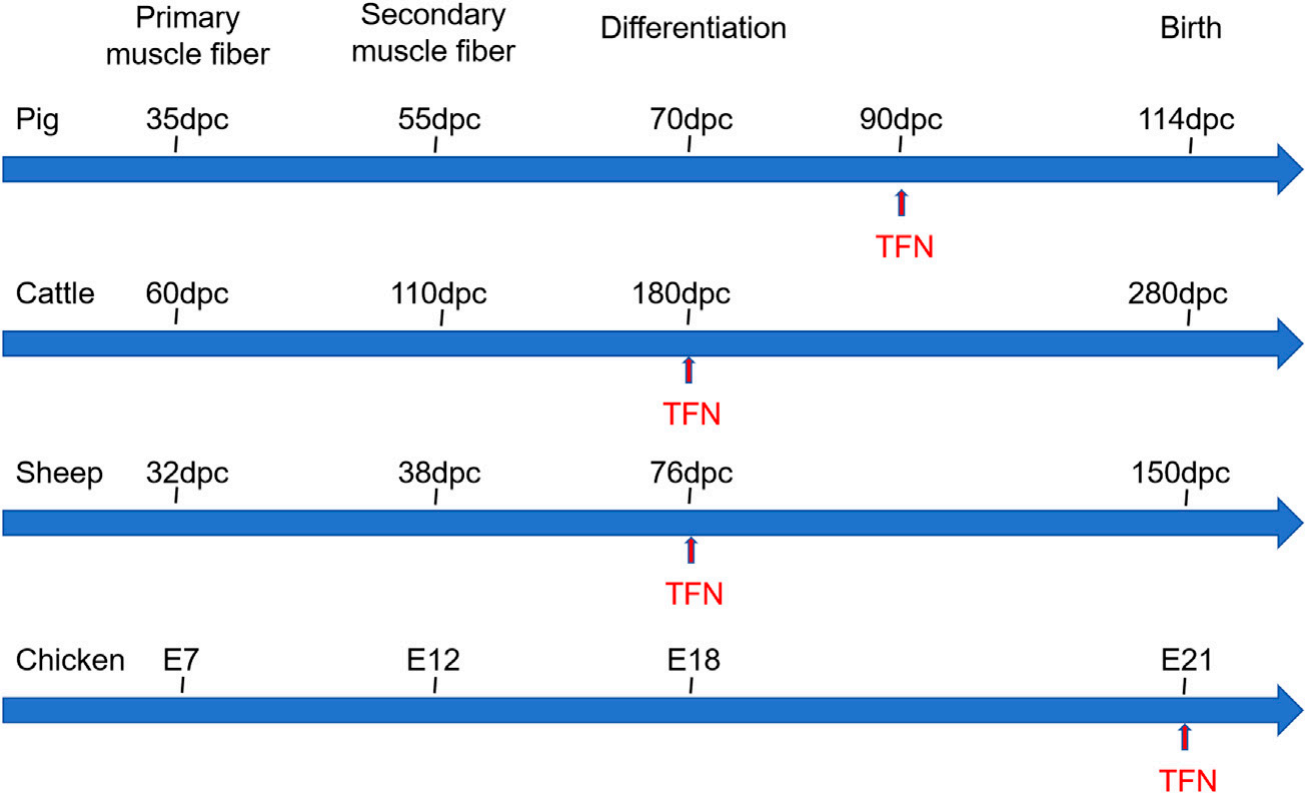
The skeletal muscle developmental processes of pig, cattle, sheep, and chicken. Dpc: day postconception, E: Embryonic days, TFN: total fiber number.

Muscle fibers, the fundamental units of muscle, determine muscle mass through their number and size. The development of skeletal muscle fibers is a complex, multi-step process that is tightly coordinated. This process can be divided into three stages: primary myogenesis, occurring during the embryonic period, where primary muscle fibers are generated; secondary myogenesis, taking place during the fetal period, marked by the formation of secondary muscle fibers around the primary fibers; and the maturation of muscle fibers post-birth. The total number of muscle fibers in an individual is primarily determined by the primary fibers, with secondary fibers forming additional muscle fibers based on the framework established by the primary ones (Gros et al., 2004). Figure 2 provides a summary of the myogenesis time for major agricultural animals such as pigs, cattle, sheep, and chickens. The two waves of muscle fiber formation during pregnancy dictate the overall number of muscle fibers (Wigmore and Stickland, 1983; Stickland and Handel, 1986), significantly influencing the growth rate and degree of postnatal skeletal muscle development (Dwyer et al., 1993). It is widely accepted that the total number of muscle fibers is fixed at birth, with the postnatal process primarily involving muscle fiber hypertrophy and maturation (Grefte et al., 2007). However, this view has been challenged by Bérard et al. (2011), who found that the total fiber number (TFN) in pig muscles is not entirely fixed at birth. Instead, they observed a postnatal increase in TFN, potentially attributed to the elongation of existing muscle fibers and the formation of tertiary fibers, primarily occurring within 3 weeks after birth. Similarly, a study (Li et al., 2015) using the Cre-loxP system to track muscle fiber formation in various skeletal muscles of mice during development revealed that the TFN in the longissimus dorsi, gastrocnemius, and rectus femoris muscles is established before birth. In contrast, the anterior tibial muscle and extensor digitorum longus exhibited different developmental patterns, with their fiber numbers continuing to increase during the first week post-birth before stabilizing. These findings suggest that the developmental dynamics of muscle fibers may vary significantly across different muscles. Given that the development and growth of skeletal muscle determine the yield and quality of meat, it is crucial to systematically understand and study the regulatory mechanisms of skeletal muscle development in agricultural animals.

The process of myogenesis is governed by a complex network of gene expression regulation, primarily facilitated through the meticulous control of intercellular signals and specific gene expression. This intricate developmental process involves multiple elements, with genetic factors playing a pivotal role. These are regulated by numerous genes, signaling pathways (Schiaffino et al., 2007), and transcription factors such as Myogenic Regulatory Factors (MRFs) (Zanou and Gailly, 2013) and Muscle Enhancer Factor 2 (MEF2) (Rullman et al., 2018), among others. Table 2 provides a summary of key regulatory factors involved in muscle fiber development. A multitude of genes contribute to the growth and development of skeletal muscle, interacting synergistically to form a complex regulatory network that ensures the stability of skeletal muscle growth and development (Braun and Gautel, 2011; Perry and Rudnick, 2000).

**TABLE 2:** Key genes and transcription factors regulating myofiber development.

Gene/Factor	Regulatory Effect	Mechanism	References
Pax3/Pax7	Maintains satellite cell population	Regulates self-renewal and activation of muscle stem cells	Relaix et al. (2005)
MyoD	Promotes myoblast determination	Activates muscle-specific gene expression	Rudnicki et al. (1993)
Myf5	Initiates myogenic program	Specifies myoblast lineage	Braun et al. (1992)
MRF4	Maintains myogenic lineage	Regulates late-stage differentiation	Zhang et al. (1995)
Myogenin	Promotes myoblast differentiation	Activates genes for terminal differentiation	Hasty et al. (1993)
Myostatin	Negative regulator of muscle growth	Inhibits myoblast proliferation and differentiation	McPherron et al. (1997), McPherron and Lee (1997)
IGF-1	Promotes muscle growth	Stimulates myoblast proliferation and differentiation	Musarò et al. (2001)
Follistatin	Promotes muscle growth	Inhibits myostatin	Lee and McPherron (2001)
FoxO1	Regulates muscle atrophy	Interacts with the PI3K/Akt pathway	Stitt et al. (2004)
MEF2	Terminal differentiation of myocytes	Activates muscle-specific gene expression	Olson et al. (1995), McKinsey et al. (2002)
Six1/4	Proliferation and migration of muscle progenitor cells	Works with Pax3/7 to regulate early myocyte development	Grifone et al. (2005)
Myomaker	Essential for myoblast fusion	Acts as a membrane protein specifically required for the fusion of muscle precursor cells into mature fibers	Millay et al. (2013)
Myomixer	Promotes myoblast fusion	Works in conjunction with Myomaker to enable myoblast membrane fusion and skeletal muscle growth	Bi et al. (2017)

In recent years, candidate genes regulating myofiber development have been continuously identified with the rapid advancement of sequencing technology and the booming development of multi-omics technology. These studies have revealed the important effects of myofiber size and number on muscle mass and yield and provided new perspectives on the molecular regulatory mechanisms of myofiber development.

The diameter of myofibers directly affects muscle mass. Wang et al. (2024) compared gene expression differences between oxidative and glycolytic muscles in bulls by transcriptome sequencing and identified 23 candidate genes (e.g., *RYR1*, *DUSP13*, and *MYH14*) that are related to myofiber diameter. Functional studies of these genes can help to reveal the molecular basis of the differences in fiber size between oxidative and glycolytic muscles. Yu et al. (2024) further constructed a transcriptome profile of skeletal muscle from 27 developmental stages from embryo to adulthood in long white pigs and identified 11 candidate genes (e.g., *CHAC1*, *RTN4IP1*, and *SESN1*), which may play a key role in the myofiber type switching and muscle volume expansion.

An increase in myofiber number is essential to enhance muscle production. A study (Nesvadbova and Borilova, 2018) in Landrace pigs identified several genes (e.g., *STMN1*, *ACVR1*, *GSK3B*, *IKBKB*, and *ITGA*) associated with increased myofiber number and regulation of late-stage muscle production. In mice, Myogenin, Klf5, and Tead4 were found to synergistically activate the transcription of genes in developing muscle (Dos Santos et al., 2023), whereas, in mature muscle fibers, the high expression of the transcription factor Maf revealed its important regulatory role in myofiber maturation. Maf was identified as a key factor in myofiber maturation by ChIP-seq experiments and analysis of Maf knockout mice.

Gene editing technology has shown considerable promise in furthering our comprehension of gene function and regulatory mechanisms. Chen et al. (2024) constructed for the first time “double muscle” sheep with double gene editing of *MSTN* and *FGF5* and revealed the molecular mechanism by which double gene editing regulates myofiber proliferation through activating the MEK-ERK pathway. The study showed that the activation of *FOSL1* could promote cell proliferation by accelerating the cell cycle transition from the G0/G1 phase to the S phase and inhibit the differentiation of skeletal muscle satellite cells by suppressing the expression of *Myod1*. In addition, *MSTN* and *FGF5* editing further mediated myofiber proliferation by inhibiting CaMKII-dependent myotube fusion. This study not only revealed the complex regulatory network of myofiber development but also bred a new breed of sheep that combines high meat yield, low fat deposition, and high-quality fine wool.

Recent advances in the molecular regulation of muscle fiber development from the above studies provide important opportunities for enhancing muscle growth and meat production in agricultural animals. The identification of species-specific regulatory mechanisms highlights the importance of studying these processes directly in agricultural animals rather than relying solely on model organism findings.

**FIGURE 3:**
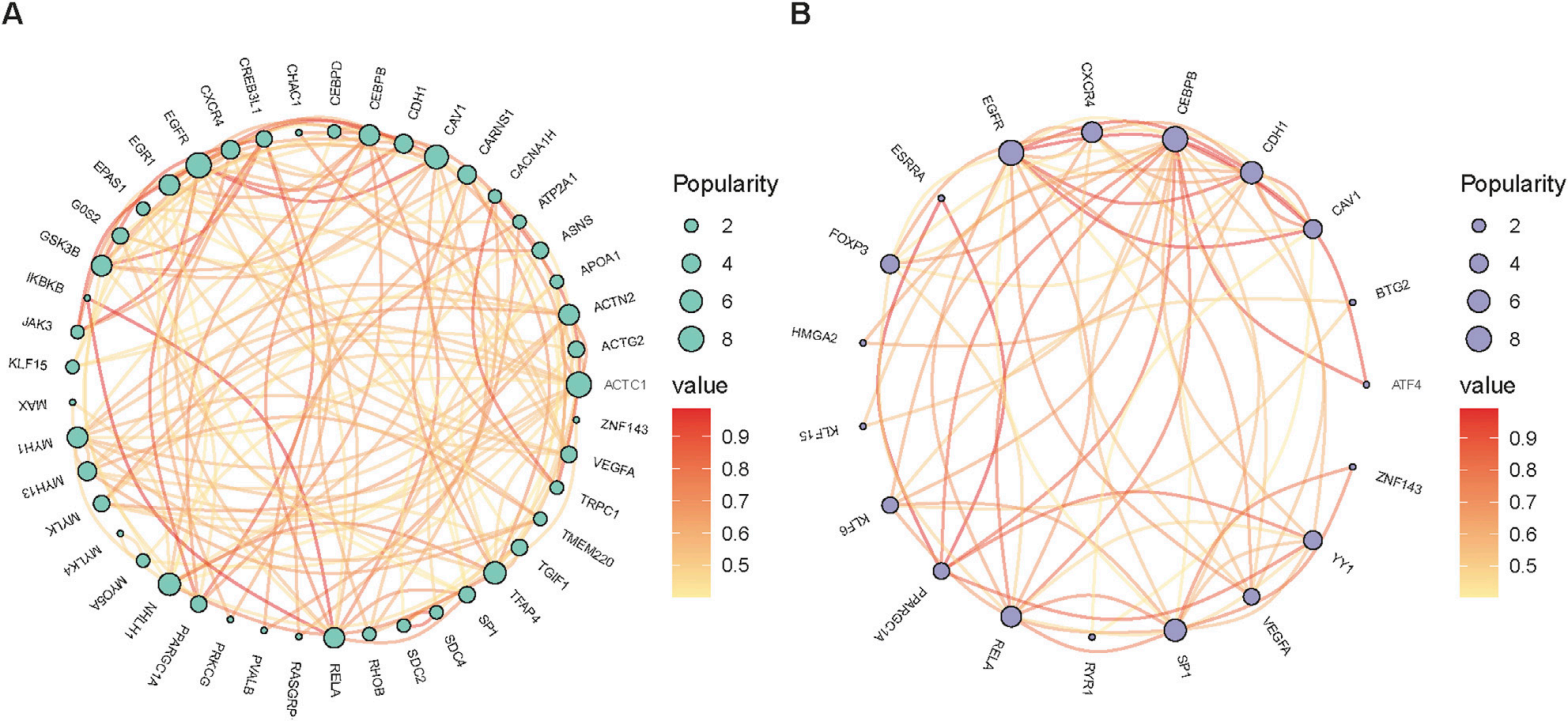
The network of candidate genes linked to muscle development in farm animals, specifically pigs **(A)** and cattle **(B)**.

To systematically summarize the findings in recent years, we have compiled a selection of new candidate genes related to muscle development in agricultural animals (Table 3) and mapped the interaction network of candidate genes related to muscle development in pigs and cattle (Figure 3). For example, YY1 is a transcription factor involved in the regulation of a variety of biological processes, including skeletal muscle development and differentiation (Chen et al., 2019). PPARGC1A is a key transcriptional co-activator that plays an important role in the regulation of energy metabolism and myofiber type transformation in skeletal muscle (Ma et al., 2022). The collaborative impact of these two genes may play an important role in myofiber differentiation, type composition, and muscle regeneration, which may provide some theoretical basis and practical guidance for further studies in the future.

**TABLE 3:** New candidate genes related to muscle development in agricultural animals.

Species	Gene	Effect	References
Pig	*CHAC1, RTN4IP1, SESN1, CHCHD3, CLNS1A, MYH2, ACTC1, ACTG2, ACTN2*	muscle fiber type	Huang et al. (2022), Yu et al. (2024), Li et al. (2024b)
	*STMN1, ACVR1, GSK3B, IKBKB, ZBTB5, MYH13, ITGA*	myofiber number	Huang et al. (2022), Nesvadbova and Borilova (2018)
	*EGR1, RHOB, ITGA7, SDC2, SDC4, AHCTF1, CEBPD, MAX, ANGTPL4, Pbx1, Znf423, Mylk, Myo5a, Mylk4, Mylk2, SAV1, CACNA1H, PRKCG, FGFR4, JAK3, SCN4A, ATP2A1, CREB3L1, TGIF1, NFIC, CEBPZ, RASGRP1, TRPC1, CEBP, TFAP4, NHLH1, SP1, PVALB, THRSP, ASNS, CARNS1, G0S2, ACBD7, TMEM220*	muscle development	Xu et al. (2023), Cai et al. (2023a), Yi et al. (2024), Zhang et al. (2019), Lan et al. (2024), Bai et al. (2023), Xiao et al. (2024), Hao et al. (2024), Xu et al. (2022), Miao et al. (2021)
Cattle	*RYR1, DUSP13, MYH14*	myofiber diameter	Wang et al. (2024)
	*MAFF, ZNF384, KLF6, HMGA2, MSC, FOXP3, ESRRA, BACH1, ATF4, Sp1, YY1, ANKRD2, ANKRD1, BTG2, LMOD3*	muscle development	Cai et al. (2023b), Cheng et al. (2023), Li et al. (2023)
Pig Cattle Sheep	*PPARGC1A, EPAS1, CXCR4, APOA1*	skeletal muscle maturation and hypertrophy	Mohammadinejad et al. (2022)
	*EGFR, VEGFA, CDH1, CAV1, CEBPB, KLF15, RELA, ZNF143, ZBTB48, REST*	muscle development	Nejad et al. (2024)
Sheep	Ythdf2*, FOXO3, PRKAG3, MYOZ2, ANKRD1, PDE3A*	muscle development	Deng et al. (2024), Deniskova et al. (2024), Li et al. (2024c)
Chicken	*ACTC1, MUSTN1, ITGB3, DNAJC27, ETV4, C7orf50, FKBP1B, G3BP1, IGF2BP1, KCNH6, LOC416263, SCARA5, SMIM5, TBL1XR1, TMEM182*	muscle development	Li et al. (2024a), Gu et al. (2024)
Duck	*TASP1*	myofiber diameter	Liu et al. (2021)

## 6 Epigenetic regulation of myogenesis

In recent years, the rapid development of epigenetics has provided significant breakthroughs in the study of muscle development and its regulatory mechanisms in agricultural animals. Epigenetic regulation affects gene expression by not altering the DNA sequence, and the main mechanisms include DNA methylation, histone modification, and non-coding RNAs, which play a key role in the growth and development of muscle fibers (Gao et al., 2017; Cao et al., 2018; Shi et al., 2015).

DNA methylation is one of the core mechanisms of epigenetic regulation, which modulates gene expression through adding methyl to the cytosine of DNA (usually occurs at CpG islands) (Baik et al., 2014; Park et al., 2018). The hypomethylated state is usually associated with gene transcriptional activity, whereas hypermethylation leads to gene silencing or expression inhibition (Moore et al., 2013; Besselink et al., 2023). For instance, differential methylation of the *cofilin-1* gene was confirmed to be closely related to the changes in myofiber formation, which has a direct impact on livestock meat quality (Sun et al., 2022). Moreover, studies (Carrió et al., 2016; Laker and Ryall, 2016) showed that myogenic stem cells exhibited an increase in DNA methylation during the proliferation stage, while the differentiation stage was accompanied by demethylation of specific gene regions, such as the enhancer region, which can activate the expression of key myogenic genes such as *MyoD* and *Myf5* (Carrio et al., 2015; Brunk et al., 1996).

Histone modification is another important means of epigenetic regulation (Zhao et al., 2007; Mikkelsen et al., 2007), which dynamically adjusts the open state of chromatin and gene expression activity through acetylation, methylation, ubiquitination, and other modifications of histone tails (Zhao and Shilatifard, 2019; Roma-Rodrigues et al., 2020). For example, H3K27me3 and H3K4me3, as key epigenetic markers in muscle development (Tan et al., 2021; Yang Y. et al., 2021), are respectively related to gene silencing and activation; histone acetyltransferase (HAT) (He et al., 2021; Yucel et al., 2019) and histone deacetylase (HDAC) (Cohen et al., 2015) play a key role in regulating muscle stem cell activation and myofiber type determination. Research has found that Taf1 regulates Pax3 protein through monoubiquitination (Boutet et al., 2010), thereby affecting the differentiation of skeletal muscle progenitor cells. The dynamic changes of these modifications determine the fate of skeletal muscle cells and the final phenotype of muscle development (Wang and Ibeagha-Awemu, 2021).

**FIGURE 4:**
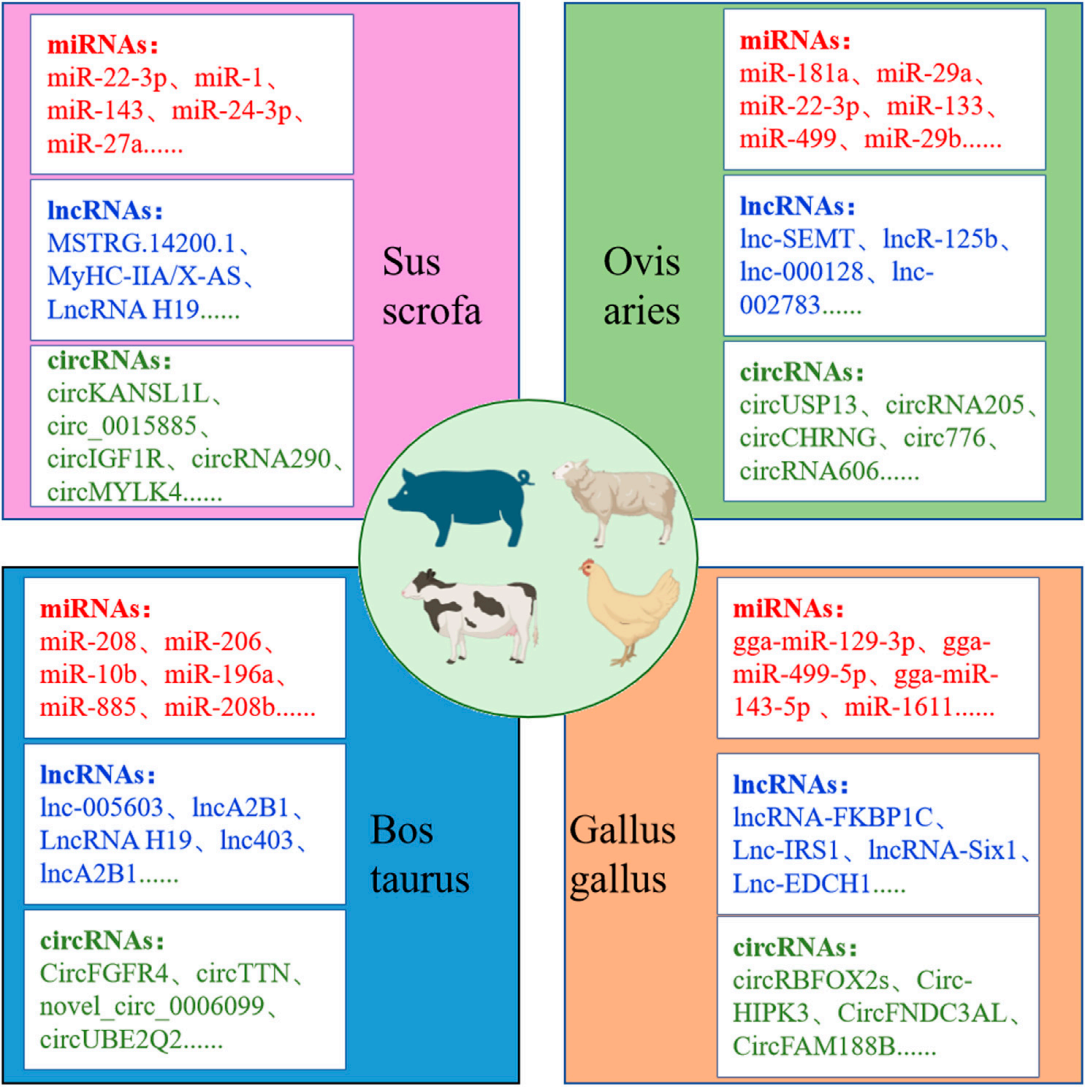
Non-coding RNAs affecting growth and development of muscle fibers in agricultural animals.

Non-coding RNAs, including miRNAs, lncRNAs, and circRNAs, play a pivotal role in muscle growth and development in agricultural animals (Huang et al., 2021; Cesana et al., 2011; Ouyang et al., 2018; Luo et al., 2013). The advent of high-throughput sequencing technology has facilitated the identification of numerous non-coding RNAs as key regulators of myofiber development. MiRNAs, for example, inhibit the expression of target genes by binding to their mRNA, thereby affecting the proliferation and differentiation of myoblasts and satellite cells. A precise study has revealed a strong correlation between the eQTL mutation site of miR-4331-5p and pig muscle fiber types (Zhong et al., 2023). LncRNAs not only influence gene expression by regulating miRNA or directly acting on gene promoters but can also encode functional short peptides to regulate muscle development. For instance (Dou et al., 2020), MyHC-IIA/X-AS, a lncRNA specifically expressed in skeletal muscle, regulates miR-130b through a competitive endogenous RNA (ceRNA) mechanism to maintain fast-twitch muscle fiber phenotypes. CircRNAs, owing to their stability and abundance, occupy a significant position in skeletal muscle (Yang Z. et al., 2021). For instance, circMEF2As has been demonstrated in studies in chickens and mice to promote satellite cell differentiation and skeletal muscle formation via the ceRNA mechanism (Shen et al., 2023). Figure 4 provides a summary of the key non-coding RNAs identified in recent years that regulate muscle development in agricultural animals, offering new molecular targets for livestock muscle trait improvement.

Intricate interactions frequently occur between various epigenetic mechanisms. For instance, non-coding RNAs can affect chromatin states by regulating DNA methylation enzymes or histone modification enzymes, and the synergy between DNA methylation and histone modification further refines the gene expression regulatory network. During muscle development, epigenetic mechanisms dynamically regulate gene expression to adapt to the developmental needs of different stages. For example, the synergy between DNA methylation and chromatin accessibility (ATAC-seq) regulates the expression of genes related to muscle hypertrophy and fiber transition in the growth process of Hu sheep skeletal muscle (Cao et al., 2023). Furthermore, research indicates that some epigenetic markers not only play a role in individual development but may also be affected by environmental factors such as parental diet or environmental exposure, thereby affecting the muscle development of offspring through transgenerational transmission mechanisms (González-Recio et al., 2015).

Advanced technologies have accelerated progress in this field. Single-cell multi-omics enables high-resolution analysis of epigenetic heterogeneity within muscle tissues, precisely tracing developmental changes (Chen et al., 2018). Integrating spatiotemporal omics data (Fomchenko et al., 2020) reveals unique epigenetic characteristics of different muscle cell populations, providing new optimization strategies.

The epigenetic insights outlined above offer significant practical applications for agricultural animal breeding and production systems. Epigenetic markers could serve as powerful selection tools in breeding programs, enabling the identification of animals predisposed to superior muscle development and meat quality traits before phenotypic expression. For example, specific DNA methylation patterns in the cofilin-1 gene could be used as early biomarkers for selecting animals with desirable myofiber characteristics. Nutritional intervention strategies targeting epigenetic mechanisms represent another promising application. Feed formulations could be designed to influence histone modifications and DNA methylation patterns during critical developmental windows, enhancing muscle growth efficiency and quality. Additionally, the identified non-coding RNAs could provide targets for novel biotechnological interventions in livestock improvement. RNA-based therapeutics or gene editing approaches targeting key regulatory non-coding RNAs might enable precise modulation of muscle fiber types to meet specific market demands for meat texture and quality. For the meat processing industry, understanding epigenetic profiles could improve processing protocols by predicting meat quality characteristics and enabling customized handling of carcasses based on their epigenetic signatures. This would reduce waste and increase value across the production chain.

In conclusion, epigenetic research is incrementally unveiling the intricate regulatory mechanisms underlying muscle development in agricultural animals, providing both theoretical frameworks and practical applications for precision breeding and meat quality optimization. As technology advances, this field will continue to drive unprecedented progress in muscle development research in agricultural animals.

## 7 Summary and future perspectives

The regulatory mechanisms of muscle fiber growth and development in agricultural animals represent critical research areas with significant implications for meat quality, yield, and economic outcomes. This review has systematically summarized heritability estimates of muscle fiber traits in agricultural animals, their effects on meat quality and yield, and the molecular and epigenetic regulatory mechanisms governing these traits.

Despite considerable progress in recent years, a substantial gap persists between muscle fiber research in agricultural animals compared to humans or model organisms. A notable deficiency is the absence of comprehensive, publicly accessible biobanks for agricultural animals, limiting analyses of relationships among phenotypes, genotypes, and environmental factors. This hinders a deeper understanding of the genetic principles underlying muscle fiber development. Additionally, while CRISPR-Cas9 technology has revolutionized gene function research, the development of CRISPR interference systems (CRISPRi) and other high-throughput screening technologies remains inadequate for agricultural animals, complicating the efficient identification of key regulatory genes and functional modules. Furthermore, cross-species comparative research on species-specific gene regulatory networks is lacking, necessitating unified databases and standardized analysis frameworks to enhance data integration.

We propose the establishment of standardized biobanks for agricultural animals as the most critical initiative for advancing muscle fiber research. This priority recommendation addresses a fundamental infrastructure gap and would catalyze progress across multiple research fronts. Launch a multi-institutional pilot biobank, focusing on economically important species such as pigs and chickens. The project will standardize the collection of genomic, transcriptomic, and phenotypic data from different breeds. Seek funding from agricultural research institutions, industry partners (e.g., meat production companies), and international partners (e.g., FAO partners). Ultimately, it creates an open-access database modeled after the human biobank, providing standardized data formats, detailed metadata, and an intuitive interface for researchers worldwide.

In summary, the exploration of muscle fiber growth and development regulation in agricultural animals is experiencing significant advancement. Bridging gaps in data and technology will catalyze the integration of emerging technologies and interdisciplinary collaborations, offering new insights into regulatory mechanisms. This will enhance meat production efficiency while contributing to global food security and sustainable development objectives.
